# Lysine Succinylation of VBS Contributes to Sclerotia Development and Aflatoxin Biosynthesis in *Aspergillus flavus*

**DOI:** 10.1016/j.mcpro.2022.100490

**Published:** 2022-12-22

**Authors:** Yu Wang, Mingkun Yang, Feng Ge, Bin Jiang, Rui Hu, Xin Zhou, Yunhuang Yang, Maili Liu

**Affiliations:** 1 State Key Laboratory of Magnetic Resonance and Atomic Molecular Physics, Key Laboratory of Magnetic Resonance in Biological Systems, National Center for Magnetic Resonance in Wuhan, Wuhan Institute of Physics and Mathematics, Innovation Academy for Precision Measurement Science and Technology, Chinese Academy of Sciences – Wuhan National Laboratory for Optoelectronics, Hubei Optics Valley Laboratory, Wuhan, China; 2 University of Chinese Academy of Sciences, Beijing, China; 3 State Key Laboratory of Freshwater Ecology and Biotechnology, Institute of Hydrobiology, Chinese Academy of Sciences, Wuhan, China

**Keywords:** *Aspergillus flavus*, lysine succinylation, quantification proteome, natural environments, aflatoxin production, AF, aflatoxin, AFB_1_, aflatoxin B1, BP, biological process, CC, cellular component, cDNA, complementary DNA, FDR, false discovery rate, GO, Gene Ontology, H-AF, high AF, KEGG, Kyoto Encyclopedia of Genes and Genomes, Ksuc, lysine succinylation, L-AF, low AF, MF, molecular function, MS, mass spectrometry, PDA, potato dextrose agar, PPI, protein–protein interaction, PTM, post-translational modification, qRT–PCR, quantitative RT–PCR, SM, secondary metabolite, TCA, tricarboxylic acid, TMT, tandem mass tag, VBS, versicolorin B synthase, YES, yeast extract with supplement, YPD, yeast peptone dextrose

## Abstract

*Aspergillus flavus* is a common saprophytic and pathogenic fungus, and its secondary metabolic pathways are one of the most highly characterized owing to its aflatoxin (AF) metabolite affecting global economic crops and human health. Different natural environments can cause significant variations in AF synthesis. Succinylation was recently identified as one of the most critical regulatory post-translational modifications affecting metabolic pathways. It is primarily reported in human cells and bacteria with few studies on fungi. Proteomic quantification of lysine succinylation (Ksuc) exploring its potential involvement in secondary metabolism regulation (including AF production) has not been performed under natural conditions in *A. flavus*. In this study, a quantification method was performed based on tandem mass tag labeling and antibody-based affinity enrichment of succinylated peptides *via* high accuracy nano-liquid chromatography with tandem mass spectrometry to explore the succinylation mechanism affecting the pathogenicity of naturally isolated *A. flavus* strains with varying toxin production. Altogether, 1240 Ksuc sites in 768 proteins were identified with 1103 sites in 685 proteins quantified. Comparing succinylated protein levels between high and low AF-producing *A. flavus* strains, bioinformatics analysis indicated that most succinylated proteins located in the AF biosynthetic pathway were downregulated, which directly affected AF synthesis. Versicolorin B synthase is a key catalytic enzyme for heterochrome B synthesis during AF synthesis. Site-directed mutagenesis and biochemical studies revealed that versicolorin B synthase succinylation is an important regulatory mechanism affecting sclerotia development and AF biosynthesis in *A. flavus*. In summary, our quantitative study of the lysine succinylome in high/low AF-producing strains revealed the role of Ksuc in regulating AF biosynthesis. We revealed novel insights into the metabolism of AF biosynthesis using naturally isolated *A. flavus* strains and identified a rich source of metabolism-related enzymes regulated by succinylation.

## Highlights

• Comparing the levels of succinylated proteins between (high/low) aflatoxin production *strains,* our succinylome data identified 1240 lysine succinylation sites in 768 proteins, among which 1103 lysine succinylation (Ksuc) sites in 685 proteins were quantified.
• Among the quantified proteins, eight Ksuc sites in eight proteins are upregulated and 502 Ksuc sites in 360 proteins are downregulated.
• Comparing WT with VBS ^K135A^ and VBS ^K135R^ strains, we further confirmed that K135 is the key succinylation site.

## Graphical Abstract

**Figure:**
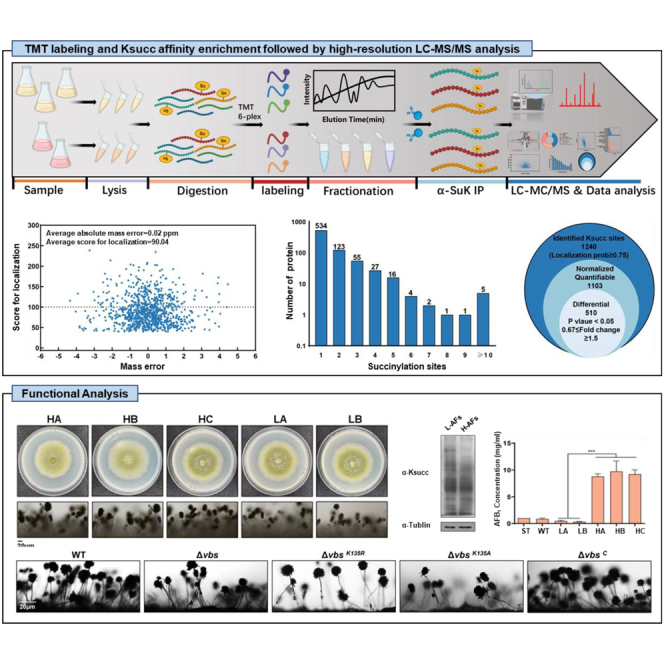

## In Brief

In this research, by comparing the succinylation levels of the two strains and exploring the relationship between succinylation and aflatoxin synthesis, our succinylome data identified 1240 lysine succinylation sites in 768 proteins, among which 1103 lysine succinylation sites in 685 proteins were quantified, the majority of proteins involved in aflatoxin biosynthetic pathway were downregulated in high aflatoxin-producing strains. In function analysis, we confirmed that K135 has a critical effect on VBS activity, maintenance of sclerotia and aflatoxin production.

*Aspergillus flavus* is a common pathogenic fungus that threatens global food safety by producing aflatoxin (AF) secondary metabolites (SMs) (1, 2). AF is a notoriously potent hepatotoxin strongly associated with liver and lung cancer, which promotes malnutrition in children (3, 4, 5). *A. flavus* is the second most common pathogen of invasive aspergillosis after *Aspergillus fumigatus* (6). It can cause human pulmonary disease as well as infestation of plants at preharvest and postharvest stages, leading to significant economic losses (7, 8, 9). Therefore, further research on AF biosynthesis and infection of *A. flavus* is imperative. *A. flavus* can be divided into several types according to its SMs and morphology (10). The differences and ecological distribution of these species are caused by regional trends and climate change fluctuations, which also reflect significant changes regulating AF biosynthesis (11, 12). Thus, exploring *A. flavus* strains with different AF yields is a useful way to study AF biosynthesis mechanisms and understand how the natural environment influences the strains.

Many natural and external environmental factors influence AF biosynthesis, including light, temperature, and humidity (12, 13, 14, 15). A more comprehensive and in-depth research on the differences in AF synthesis under natural conditions is essential for developing effective methods to eliminate or reduce AF contamination in food supply (16). In recent decades, the *A. flavus* genome was sequenced, and the AF biosynthetic pathway was investigated in detail (17, 18, 19, 20). This pathway consists of a complex set of enzymatic reactions, including the catalysis of acetyl-CoA and malonyl-CoA by Fas-1 and Fas-2, production of versicolorin B intermediate by versicolorin B synthase (VBS), and eventual synthesis of AFs B_1_ and B_2_ (21, 22, 23). However, genome annotation and transcriptome differences cannot directly explain the relationship between the SMs of *A. flavus* and proteins involved in AF synthesis (24). Moreover, enzymatic reactions in AF synthesis ultimately depend on their activity (25). Nowadays, mass spectrometry (MS)–based proteomics are commonly used for high-throughput protein analysis and identification (26). For example, *A. flavus* proteomics profiling showed that laeA regulates AF biosynthesis by influencing extracellular hydrolases and conidial hydrophobin and responding to oxidative stress (27). Quantitative proteomic analysis of *A. flavus* revealed that the Ras subfamily GTPases are crucial for AF biosynthesis and pathogenicity (28). Therefore, proteomics is an effective technique to study the intricate molecular mechanism underlying AF biosynthesis.

Protein post-translational modifications (PTMs) represent an important modulating method for proteome diversification to regulate cellular processes (29, 30, 31). Covalent modification by introducing amino acids modulates protein function by affecting protein structural conformation, activity, stability, and cellular positioning (32). Lysine side chains of proteins undergo the most frequent PTM, including acetylation, lactylation, propionylation, malonylation, butyrylation, crotonylation, succinylation, 2-hydroxyisobutyrylation, glutarylation, long-chain fatty acylation, methylation, phosphorylation, and ubiquitination (33, 34, 35, 36, 37, 38, 39). Acyl-CoA metabolites directly influence AF biosynthesis in *A. flavus* (40). Lysine succinylation (Ksuc) is a key modification for regulating enzyme activity related to metabolite synthesis (41). Protein Ksuc is formed by transferring the succinyl group (-CO-CH_2_-CH_2_-CO_2_H) to the lysine residue of the protein (42). Ksuc introduces a relatively large group, unlike methylation and acetylation (43). Though, it produces a two-unit charge shift by neutralizing the positive charge and producing a net negative charge in the modified residue (44). Many recent reports have established that Ksuc is involved in regulating enzyme activity in various metabolic pathways (45, 46). However, there are few quantitative studies measuring Ksuc levels under different AF yields in natural conditions. Further research is required to determine whether changes in the Ksuc level under natural conditions influence metabolic pathways involving toxin production in *A. flavus*.

In this study, a quantification method was performed based on tandem mass tag (TMT) labeling and antibody-based affinity enrichment of succinylated peptides, followed by quantitative identification of Ksuc sites in naturally isolated *A. flavus* strains with varying toxin production. In addition, we primarily focused on the metabolic activities affecting AF production related to Ksuc levels. Comparative bioinformatic analysis of succinylation in these naturally high and low AF-yielding strains (H-AF and L-AF, respectively) revealed that most of the enzymes involved in *A. flavus* metabolism were modified, and the level of modification was significantly different. To obtain comprehensive insights into succinylation and AF synthesis, we performed a functional analysis of Ksuc sites on VBS, a key enzyme for AF synthesis. The modification level was significantly different in H-AF and L-AF yields. We found that mutations in the Ksuc sites of VBS reduced aflatoxin B_1_ (AFB_1_) synthesis. In summary, we described the global protein succinylation quantitative modification analysis of natural isolates with H-AF and L-AF production and revealed that succinylation levels greatly regulated AF synthesis.

## Experimental Procedures

### *A. flavus* Strains, Media, and Culture Conditions

Naturally isolated high (HA, HB, and HC) and low (LA and LB) AF-yielding *A. flavus* strains were obtained from the Oil Crops Research Institute of the Chinese Academy of Agricultural Sciences. The *A. flavus* strain NRRL 3357 (WT) was obtained from Sun Yat-Sen University. All strains were inoculated onto potato dextrose agar (PDA; BD Difco) and yeast extract with supplement (YES) solid media (20 g/l yeast extract, 150 g/l sucrose, and 1 g/l MgSO_4_•7H_2_O).

### AF Preparation and Quantification

About 10^6^ conidia was inoculated into 10 ml YES liquid medium and cultivated in the dark at 29 °C for 7 days. Next, AF was prepared by adding 2 ml of chloroform to the liquid culture. Then, the AF-containing organic phase was transferred to a new centrifuge tube, dried completely at 70 °C, and resuspended with 100 μl of chloroform. TLC was used to separate and detect the content of AFB_1_, and 2 to 5 μl solution was loaded onto a silica gel plate (GF254; Qingdao Haiyang Chemical Industry), and solvent was separated (chloroform:acetone = 9:1). After that, AFB_1_ was detected on the plate by exposure to UV light (365 nm).

LC–MS was used for quantification of AF content; conditions were as described in our previous work by Yang *et al.* (47). The HPLC system using an analytical C18-nanocapillary LC column (1.7 μm particle, 2.1 × 50 mm) was used to separate, and the linear gradient elution program was carried out as the previous study at 200 nl/min flow rate. The MS was operated in selected reaction monitoring mode, and the precursor ions of AFs AFB_1_/AFB_2_ were set to 312.92/314.78 *m/z*. The MS source conditions were set as follows: cone voltage set to 30 V, capillary voltage set to 3 kV, collision energy set to 35 V, nitrogen gas flow rate at 50 l/h, source temperature at 150 °C, and the desolvation temperature at 350 °C. Data were acquired using MassLynx (Waters) 4.0.

### Cell Lysis and Protein Extraction

In this study, all protein samples were obtained from our previous global proteomics protein preparation and prepared from a single experiment (48). In detail, 10^6^ conidia was inoculated into liquid YES media and shaken at 180 revolutions/min for 5 days at 28 °C. Then, the harvested growing mycelia were ground using liquid nitrogen and resuspended in radioimmunoprecipitation assay lysis buffer (Beyotime). The homogenized lysate was rotated at 4 °C for 1 h, and after spinning at 8000*g* for 20 min at 4 °C, the soluble protein was transferred to a new Millipore Amicon Ultra-15 centrifugal filter (Sigma) for further removal of other small molecules and pigments. Bicinchoninic acid protein assay kit (Tiangen) was used to determine the concentration of extracted protein.

### In-Solution Trypsin Digestion, TMT Labeling, and HPLC Fractionation

The whole lysate was precipitated using 10% TFA and 1% sodium deoxycholate and then desalted thrice with ice-cold acetone. After washing, approximately 2 mg of the dried protein pellet was redissolved in 50 mM NH_3_HCO_3_. The protein extracts were reduced by adding 25 mM dl-dithiothreitol for 45 min at 37 °C. Alkylation was performed by adding 50 mM iodoacetamide at 25 °C for 30 min in the dark. For in-solution trypsin digestion, the protein solution was trypsinized in a 1:50 trypsin-to-protein mass ratio at 37 °C for 12 h. Furthermore, for complete digestion, trypsin was added in a 1:100 trypsin-to-protein mass ratio at 37 °C for 4 h. After trypsin digestion, enzymatic hydrolysis was quenched by adding 0.1% TFA. The peptide solutions were desalted by a Strata X C18 SPE column (Phenomenex) and vacuum dried; the resulting pellets were reconstituted in 0.5 M triethylammonium bicarbonate, and the peptides were labeled with a 6-plex TMT kit for global proteome and lysine succinylome quantification (49). Briefly, 100 μg of peptides from each sample were labeled with one unit of TMT reagent, mixed together, and then reconstituted in acetonitrile solution. The resulting peptide mixture was incubated at 25 °C for 2 h and then desalted and vacuum dried again. We then collected the sample at 1 min/tube from the 22nd to 78th tubes. The peptides were combined into six fractions and dried using a vacuum centrifuge. The sample was fractionated using HPLC with the Agilent 300 Extend C_18_ column (5 μm particles, 4.6 mm inner diameter, 250 mm length) (50).

### Immunoaffinity Enrichment and LC–MS/MS Analysis of Succinylated Peptides

Ksuc peptides were enriched using prewashed antibody beads (PTM Biolabs, Inc), as previously described (51). Briefly, the tryptic peptides were dissolved in NETN buffer and then added to antisuccinyl beads for incubation at 4 °C overnight with gentle rotation. Finally, the bound Ksuc peptides were eluted with 0.1% TFA and then vacuum dried. Before LC–MS/MS analysis, Ksuc peptides were desalted with C_18_ ZipTips (Millipore), vacuum dried, and stored at −80 °C.

For LC–MS/MS, the peptides were redissolved in 0.1% formic acid, and the solution was directly loaded onto a reversed-phase precolumn (Acclaim PepMap 100; Thermo). The peptides were then separated by a reversed-phase analytical column (Acclaim PepMap RSLC; Thermo). Subsequently, on an EASY-nLC 1000 UPLC system, the gradient included an increase in solvent B (0.1% formic acid in 98% acetonitrile) from 7% to 25% for 24 min, 25% to 40% for 8 min, which then rose to 80% in 5 min, and then stayed at 80% for the last 3 min, at a constant flow rate of 400 nl/min.

### Database Search and Protein Quantification

MS/MS data from LC–MS/MS were processed using MaxQuant with the integrated Andromeda search engine (version 1.5.2.8, Max Planck Institute of Biochemistry) (52). Tandem mass spectra were searched against the *A. flavus* protein database downloaded from the National Center for Biotechnology Information (http://www.ncbi.nlm.nih.gov/, released in 2013, containing 13,485 protein sequences) and concatenated with a reverse decoy database. The mass errors of the precursor ion and fragment ion were set at 10 ppm and 0.02 Da, respectively. Trypsin/P was designated as the cleavage enzyme, allowing for up to four missing cleavages, with five modifications and five charges per peptide. Carbamidomethylation on Cys was specified as a fixed modification, with succinylation on Lys, oxidation on Met, and acetylation (protein N-terminal) set as variable modifications. The estimated false discovery rate (FDR) thresholds for the modification site, peptide, and protein were set at 1%, with the minimum peptide length set to 7. For quantification, TMT-6-plex was used, and all other parameters in MaxQuant (version 1.5.2.8) were set to default values. The raw LC–MS dataset intensity was first normalized by the median value. To further quantify the relative abundance of Ksuc sites, they were normalized against their corresponding protein expression levels, and the effect of protein expression on modification abundance was removed. In the first step, the mean of the ratios of three replicates was taken as the final differentially modified relative quantification (ratio) of the comparison group. The second step used the one-sample *t*-test to calculate the *p* value of the differential modification. When *p* value <0.05 and protein ratio >1.5, the change in differential modification was regarded as significant upregulation; however, when *p* value <0.05 and protein ratio <1/1.5, the change was regarded as significant downregulation. All MS data obtained were deposited in the ProteomeXchange repository, Respectively, the global proteomics and lysine succinylome datasets for the two strains in this study can be found in ProteomeXchange datasets PXD027517 and PXD038357 (username: reviewer_pxd031353@ebi.ac.uk; password: 9q046jup) (51).

### Bioinformatics Analysis

Based on the Gene Ontology (GO) annotation of the Blast2GO (Biobam) software, the identified proteins were classified into biological process (BP), cellular component (CC), and molecular function (MF). The online DAVID Functional Annotation Bioinformatics Microarray Analysis (ncifcrf.gov) was used for GO term function enrichment analysis, and the Kyoto Encyclopedia of Genes and Genomes (KEGG; http://www.genome.jp/kegg/) database was used to annotate the protein pathway. Statistical significance was analyzed using the hypergeometric test and expressed as a *p* value. *p* < 0.05 was considered to be statistically significant. Subcellular localization of the succinylated proteins was predicted using wolfpsort (http://wolfpsort.seq.cbrc.jp/). A sequence model comprising modify-21-mers at specific positions (10 amino acids upstream and downstream of the site) was analyzed using Motif-X (https://motif-x.med.harvard.edu/motif-x.html) website. All succinylated proteins were analyzed for secondary structure prediction using NetSurfP (NetSurfP - 2.0 - Services - DTU Health Tech) website. Protein–protein interaction (PPI) information was retrieved from STRING (https://string-db.org/) and visualized using Cytoscape software (version 2.8.3, Institute for Systems Biology). A phylogenetic tree was constructed using MEGA (version 6.0, Mega Limited, Auckland, New Zealand), and the protein domain functional description was annotated using InterProScan (InterPro [https://www.ebi.ac.uk/interpro/]).

### SDS-PAGE and Immunoblotting Analysis

The equal amounts of protein extracts (60 μg) were subjected to 12% SDS-PAGE and electrotransferred from SDS-gel to polyvinylidene difluoride membrane (GE Healthcare). After that, the membrane was blocked with 5% (w/v) bovine serum albumin in Tris-buffered saline with Tween-20 at ambient temperature for 2 h, and then succinyllysine polyclonal rabbit antibody (1:1000 dilution; PTM Biolabs, Inc) was added at 4 °C overnight, following the primary antibodies, washing the membrane with Tris-buffered saline with Tween-20 (25 mM Tris–HCl, pH 8.0, 125 mM NaCl, and 0.1% Tween-20), 15 min for three times. The membrane was incubated with horseradish peroxidase–conjugated anti-rabbit immunoglobulin G (1:2000 dilution) (Abgent) for 2 h at room temperature. Finally, the chemiluminescence of immune complexes was detected by using G:BOX Chemi XT4 system (Syngene).

### VBS Deletion and Site Mutation Strain Construction

For functional analysis, the *A. flavus* strains (Δ*vbs*, Δ*vbsK*^C^, Δ*vbsK*^135A^, and Δ*vbs*^K135R^) were constructed through *vbs* gene deletion/point mutants and complemented with the method and detection based on previous studies (53, 54, 55). All fragments had primers on both ends to introduce homologous fragments. The fused amplification products were transferred to the protoplasts (*A. flavus* CA14 PTs strain). The *vbs* gene site mutation K135A/R strains were selected on YES plates without uracil and then detected *via* DNA sequencing analysis.

For the complemented strain Δ*vbs*^*C*^, the fragment (containing the upstream promoter element and 3′-nontranslated region) was amplified from *A. flavus*, and the primers containing homology arms on both sides of HindIII and KpnI were located at the restriction site of pPTR I (Takara) (supplemental Table S1), the amplification product was fused into the double restriction (HindIII and KpnI) enzyme vector, and the constructed plasmid (pPTR-*vbs*) was transformed into the protoplasts (Δ*vbs*). Finally, Δ*vbs*^*C*^ strains were screened on YES plates containing pyrithiamine.

For site mutation Δ*vbsK*^135A^ and Δ*vbs*^K135R^ strains, the four fragments (1707bp-5′UTR of *vbs to vbs* K135A/R, 1627*bp-vbs* K135A/R to *vbs* terminator, 1890 pyrG, and 1446bp-3′UTR of *vbs*) were amplified and fused by overlap PCR with the primers (M-overlap) (supplemental Table S1), and all fragments have primers on both ends to introduce homologous fragments. The fused amplification product was transferred into the protoplasts (*A. flavus CA14 PTs* strain), and *vbs gene site mutation* K135A/R strains were selected on YES plates without uracil, and then detected by DNA sequencing analysis.

### Growth, Conidia, and Sclerotia Production Analysis

To analyze strain growth, conidia formation, and sclerotia development, the colony diameter, conidia, and sclerotia were measured and counted as previous studies (56). About 10^6^ spores of different strains were inoculated on YES, PDA, and yeast peptone dextrose (YPD) plates, respectively, and grown at 37 °C. Cultured in the dark for 4 days, the strains inoculated on YES and PDA solid medium were used to record the colony morphology, and the number of conidia was counted with a hemocytometer. The strains were inoculated on YES plates in the dark for 2 days for observation of conidiophores. For sclerotia formation analysis, spores were inoculated on YPD plates at 37 ^◦^C in the dark for 7 days, the sclerotia on medium were counted, and then the sclerotia morphology on the YPD plates after washing with 75% ethanol was recorded. All experiments were repeated three times independently with three biological replicates. All experiments were repeated three times independently with three biological replicates.

### Pathogenicity Assay

Pathogenicity assays were measured as described previously (57). Briefly, peanut cotyledons were sterilized by rinsing three times with 0.05% sodium hypochlorite and 75% ethanol and then washed five times with sterile water to remove reagent residues. After sterilization, these peanut cotyledons were inoculated with 10^6^ spores of WT and mutant strains 1 h with continuous shaking at 50 rpm, and the mock was performed by inoculating sterile water. Subsequently, the infected peanut cotyledons were transferred into new dishes with moist sterile filter paper at 28 °C in the dark for 5 days, and then the infected peanut cotyledons were collected into 50 ml tubes; 15 ml of 0.05% Tween-80 was added to quantify conidia, and an equal amount of chloroform was added to prepare AF production as previously described (56). The pathogenicity assay was repeated at least three times.

### Quantitative RT–PCR Analysis

For quantitative real-time PCR analysis, the mycelia at growth stages were harvested, and total RNA was prepared with TRIzol reagent (Biomarker Technologies), and complementary DNA (cDNA) was synthesized with the First Strand cDNA Synthesis Kit (TransGen Biotech), and then the synthesized cDNA was diluted 10-fold as a quantitative RT–PCR (qRT–PCR) template. The SYBR Green qPCR mix (Takara) was used for qRT–PCR amplification in PikoReal Real-Time PCR machine (Thermo Scientific), and *actin* gene was used as an endogenous control. The 2^−ΔΔCT^ method was used for all target gene expression calculation and all primers listed in supplemental Table S2, and all qRT–PCR experiments were repeated three times.

### Experimental Design and Statistical Rationale

To identify the succinylation-regulated substrates affecting AF synthetic yield, a quantitative lysine succinylome analysis was performed by comparing the H-AF and L-AF-yielding *A. flavus* strains. In this study, all protein samples were obtained from previous global proteomics protein preparation (51). In detail, three biological samples each of H-AF and L-AF-yielding *A. flavus* strains were harvested and digested, after which TMT labeling and antibody-based affinity enrichment were performed for LC–MS/MS analysis. The resulting MS/MS data were processed using MaxQuant (version 1.5.2.8) with integrated Andromeda search engine (version 1.5.2.8). Tandem mass spectra were searched against *A. flavus* database concatenated with reverse decoy database. The FDR thresholds for protein, peptide, and modification sites were specified at 1%. DAVID was used for the GO term function enrichment analysis. Statistical significance was analyzed using the hypergeometric test and set at *p* < 0.05. In all succinylated peptides, the sequence model comprising modify-21-mers at specific positions (10 amino acids upstream and downstream of the site) was analyzed using Motif-X with a significance level of 0.000001, and all succinylated proteins were analyzed for secondary structure prediction using NetSurfP website, with the *p* value calculated using the Wilcoxon test.

In the functional study, all experiments were designed with triplicates of biological samples: WT, *Δvbs*^*K135A*^, *Δvbs*^*K135R*^, *Δvbs*, and *Δvbs*^*KC*^. All mutant strains were constructed and verified *via* PCR and sequencing analysis. Western blot was used to analyze growth, conidia, and sclerotia production. qRT–PCR and pathogenicity assay were performed on WT, *Δvbs*, *Δvbs*^*KC*^ and the site-directed mutant *Δvbs*^*K135A*^, *Δvbs*^*K135R*^, and all data were presented as mean ± standard deviation. GraphPad Prism 8 software (GraphPad Software, Inc) was used for data statistics and significance analysis, *p* < 0.05 was considered significant difference, and the difference between the Student’s *t* test was used, and the Tukey’s multiple comparison test was used for multiple comparison analysis. Standard errors of three replicates were represented using error bars.

## Results

### Succinylation Differences Between H-AF and L-AF-Yielding *A. flavus* Strains

**Fig. 1:**
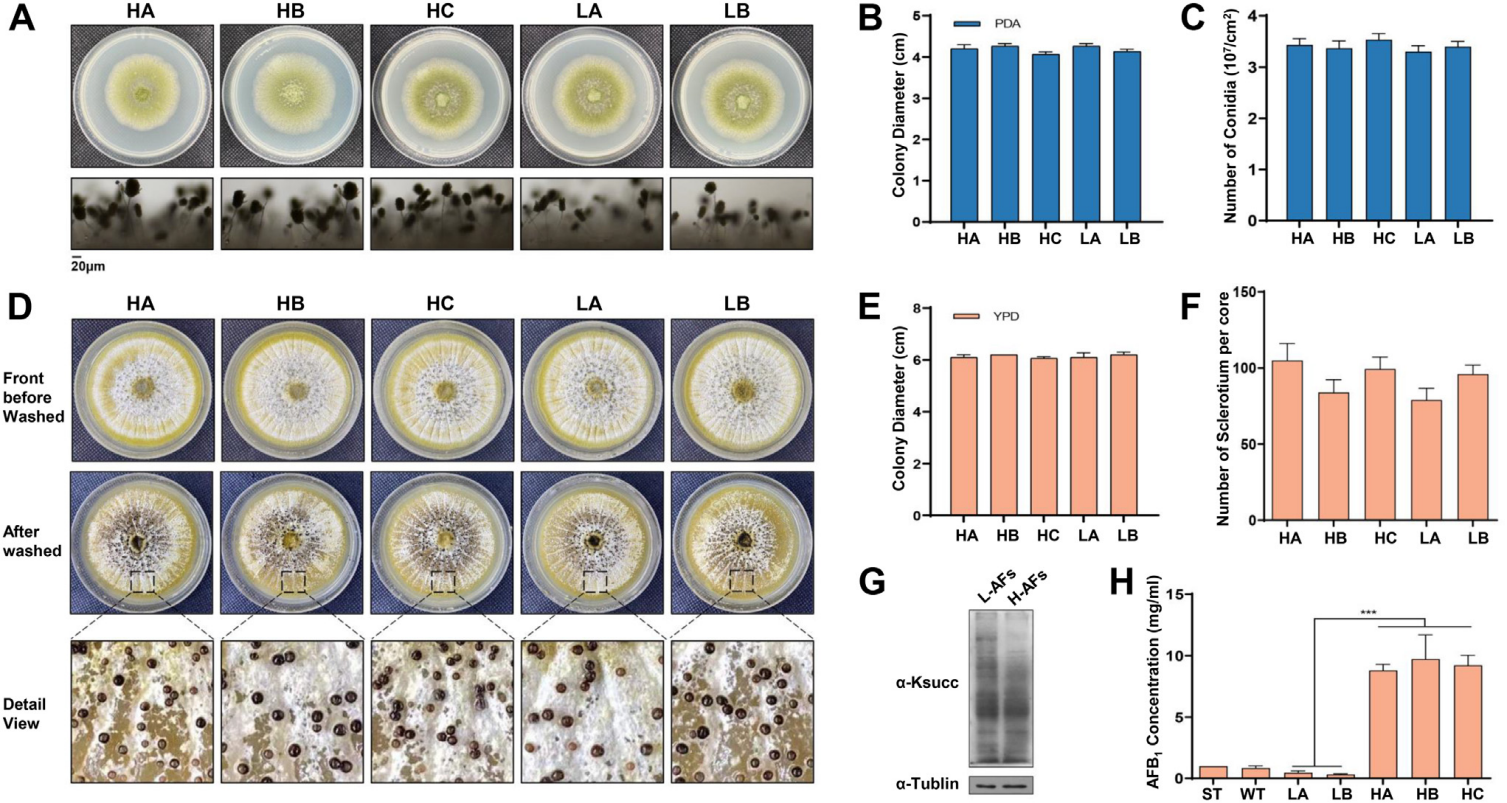
**Phenotypes of high and low AF-production *Aspergillus flavus* strains.***A*, colony morphology and microscopic observation of AF-HA, AF-HB, AF-HC, AF-LA, and AF-LB strains cultured on plates at 37 °C for 3 days. *B*, growth assays of five strains on PDA plates. *C*, quantitative analysis of the conidial amounts of five strains. *D*, five strains inoculated on YPD plate at 37 °C for 7 days, the sclerotia were observed before or after washed by 75% ethanol. *E*, growth assays of five strains on YPD plates. *F*, quantitative analysis of the sclerotia amounts of five strains. *G*, the concentration of AFB_1_ was assessed by LC–MS. The *asterisks* ∗∗∗ represents a significant difference level of *p* < 0.001. *H*, Western blot analysis of lysine succinylation in high and low AF-production *A. flavus* strains. AF, aflatoxin; AFB_1_, aflatoxin B_1_; PDA, potato dextrose agar; YPD, yeast peptone dextrose.

**Supplemental Figure S1:**
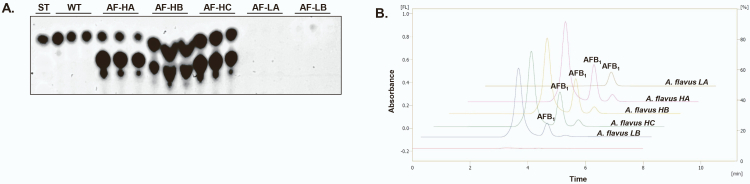
**TLC and high-performance liquid chromatography analysis of aflatoxin production in the five different strains, which were cultured in PDB medium at 28 °C for 5 days**.

Five naturally isolated *A. flavus* strains with different AF yields were obtained from peanut field soils in Oil Crops Research Institute of the Chinese Academy of Agricultural Sciences. Their phenotypes were almost identical during growth and conidia development based on the number of spores, conidia, and sclerotia when grown on PDA and YPD plates (Fig. 1). TLC analysis of the same number of spores inoculated on YES liquid medium under the same conditions showed that *A. flavus* HA, *A. flavus* HB, and *A. flavus* HC strains had significantly higher AFB_1_ production than *A. flavus* LA and *A. flavus* LB (supplemental Fig. S1). AF production by *A. flavus* HA, *A. flavus* HB, and *A. flavus* HC strains was 10-fold higher compared with *A. flavus* LA and *A. flavus* LB using HPLC coupled to a Xevo TQ MS instrument (Fig. 1*G*).

Western blot results showed that H-AFs showed a much lower number of bands than the L-AFs based on the antisuccinyllysine antibody (Fig. 1*H*). These results indicated that the level of Ksuc is significantly related to metabolism and may regulate AF production whilst having a minor effect on conidia and sclerotia growth.

### LC–MS/MS Analysis of the Succinylome in Naturally Isolated *A. flavus* Strains

**Fig. 2:**
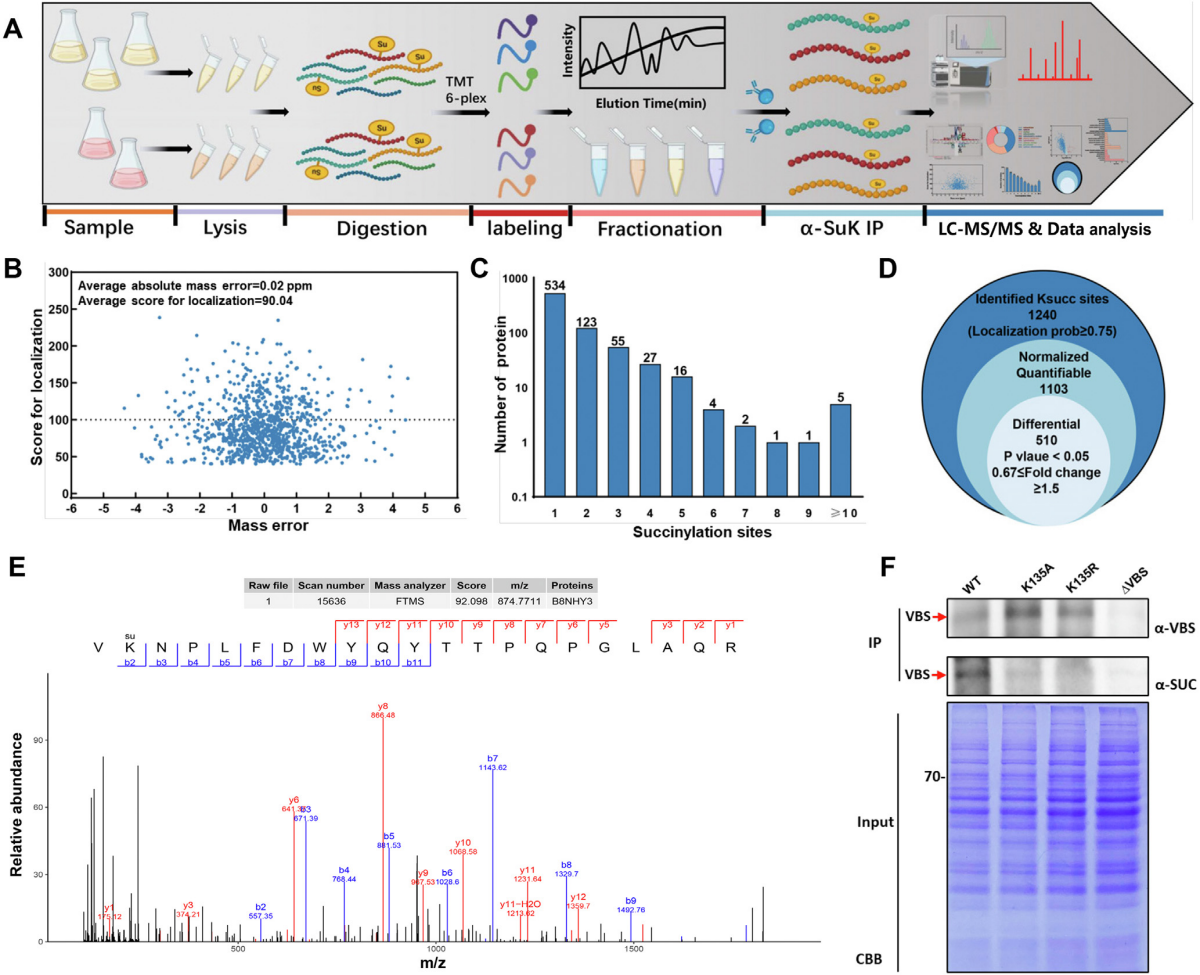
**Profiling of tandem mass tag (TMT)-based quantitative proteomics in high- and low-AF yielding *Aspergillus flavus* strains.***A*, workflow: experimental approach used to identify succinylated peptides. *B*, distribution of the lysine-succinylated peptides. *C*, histogram of the number of Ksuc sites per protein. *D*, Venn diagram showing the total numbers of Ksuc sites identified in high/low AF production strains. *E*, a representative MS/MS spectrum of a succinylated peptide from the VBS. *F*, Western blotting analysis of immunoprecipitated VBS. AF, aflatoxin; Ksuc, lysine succinylation; VBS, versicolorin B synthase.

**Fig. 3:**
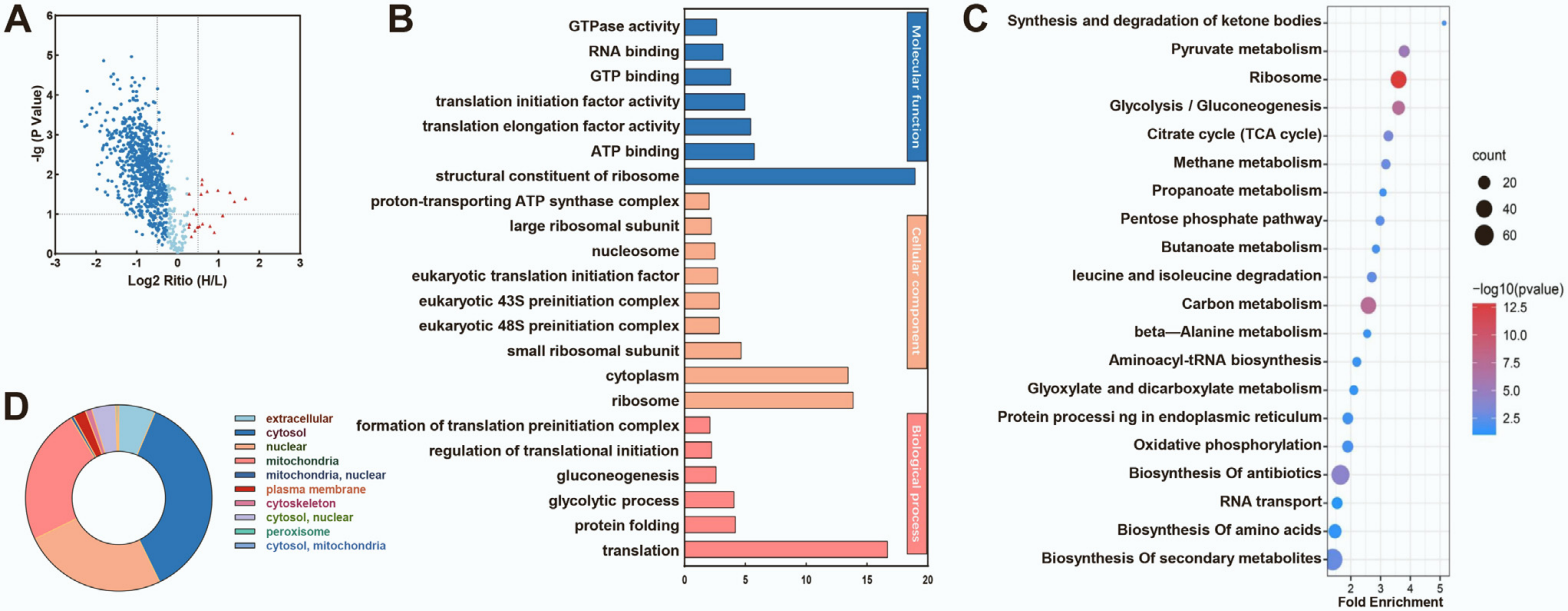
**Tandem mass tag (TMT)–based quantitative proteomic analysis of lysine succinylation proteome.***A*, Volcano map showing the quantification of lysine succinylation sites in relation to peptide intensities. *B*, functional analysis of the succinylated proteins, the identified succinylated proteins enrichment of Gene Ontology (GO) categories for biological processes, molecular functions, and cellular components were performed using DAVID bioinformatics tools (*p* < 0.05). *C*, Kyoto Encyclopedia of Genes and Genomes (KEGG) enrichment clustering showed by bubble chart. *D*, subcellular localization of identified succinylated proteins.

**Supplemental Figure S2:**
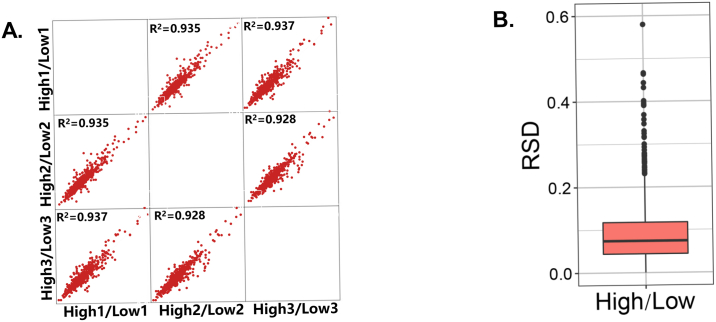
**Reproducibility and accuracy of the quantitative proteomic analysis on.***A*, Pearson correlation coefficient for three repeated experiments. *B*, RSD (relative standard deviation) analysis of TMT labeling quantification proteomic.

Two sets of large-scale proteomic analysis from H-AFs and L-AFs were performed using immunoaffinity enrichment to identify and quantify specific Ksuc proteins and sites regulated when AF production significantly differs (Fig. 2*A*). The overall average absolute peptide mass error was 0.02 ppm, and the average peptide score for localization was 90.04 (Fig. 2*B*) with most proteins having a single Ksuc site, whereas several others exhibiting multiple modifications (Fig. 2*C*). In total, 1240 Ksuc sites in 768 proteins were identified, among which 1103 Ksuc sites in 685 proteins were quantified (Fig. 2*D*) with an estimated FDR of below 1%. The reproducibility analysis of Pearson’s correlation coefficient for three repeated experiments is shown in supplemental Fig. S2. Quantification analysis involved removing the expression difference of the global proteome between H-AFs/L-AFs (supplemental Table S3). Figure 2*E* shows the MS/MS spectrum of the succinylated peptide from VBS (UniProt ID: B8NHY3). In addition, the overall absolute mass accuracy for the peptides was 0.75162 ppm, and the peptide score was 92.098 (Fig. 2*B*), further confirming the reliability and high accuracy of the succinylated peptide data obtained from MS. In order to further confirm that the K135 of VBS was the potential site for succinylation, Figure 2*F* shows that the succinyl-modified immunoblot signal of the VBS point mutant (K135A, 135R) protein that has been immunoprecipitated is significantly decreased. A comparison of differentially expressed proteins between H-AF and L-AF strains showed that eight Ksuc sites in 315 proteins were upregulated (quantitative ratio ≥ 1.5, *p* < 0.05) and 502 Ksuc sites in 360 proteins were significantly downregulated (quantitative ratio ≤0.667, *p* < 0.05) (Fig. 3*A*).

The detailed information of all identified succinylated peptides is provided in Supplemental Table S4. There were more succinyl-modified proteins showing relatively lower expression levels in H-AFs, compared with L-AFs. Over 45% sites were downregulated in H-AFs showing that Ksuc levels may significantly impact AF production and suggesting that Ksuc is involved in mediating an important pathway for SM synthesis in *A. flavus* and negatively correlates with AF synthesis. These differences in Ksuc expression and AF synthesis need further explanation by functional analysis.

### Functional Annotation and Cellular Localization of *A. flavus* Succinylated Proteins

The 502 blue dots and eight red dots in the Volcano map indicate the downregulated and upregulated differentially expressed Ksuc sites in H-AFs/L-AFs, respectively (Fig. 3*A*).

**Supplemental Figure S3:**
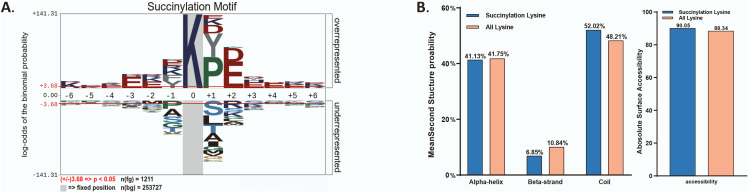
**Analysis of Lys-succinylation.***A*, sequence properties of Lys-succinylation sites in high/low aflatoxin production strains. *B*, probable localization Lys-succinylation and all lysine in different secondary structures (α-helix, beta-strand, and coil) and protein surface.

The GO functional classification of all Ksuc proteins based on their BP, CC, and MF was investigated using Blast2GO to better understand the succinylome differences in AF synthesis (supplemental Fig. S3 and supplemental Tables S5–S7). Amongst the eight upregulated Ksuc proteins, two were related to localization in BP, three were related to catalytic activity in MF, and three were related to the membrane in CC according to the H-AF/L-AF classification (supplemental Fig. S3*A* and supplemental Table S5). Downregulated Ksuc proteins were mostly associated with metabolic processes (202 or 34%) including 172 (28%) related to cellular processes in BP and 133 (22%) located in single-organism processes. In MF, most Ksuc proteins were involved in binding (203 or 44%), catalytic activity (197 or 43%), structural molecule activity (32 or 7%), and transporter activity (12 or 3%), with 41% of downregulated Ksuc proteins located in the intracellular fraction and 24% in organelles (supplemental Fig. S3*B* and supplemental Table S6). Details of the functional classification of differentially quantified proteins are stated in supplemental Fig. S3*C* and supplemental Table S7.

Compared with eight upregulated proteins, the distribution of downregulated proteins in GO terms of level 2 further suggests that the downregulated Ksuc proteins were more importantly related in metabolism. Proteins and quantified succinylation sites for BPs, CCs, and MFs were clustered using GO enrichment (supplemental Table S7). Downregulated Ksuc proteins in BPs were significantly enriched in translation (1.97e^−17^), protein folding (6.39e^−05^), and glycolytic process (8.39e^−05^). The MFs of succinylated proteins included the structural constituent of the ribosome (1.07e^−19^), ATP binding (1.80e^−06^), translation elongation factor activity (3.52e^−06^), translation initiation factor activity (1.07e^−05^), GTP binding (1.56e^−04^), and RNA binding (6.83e^−04^). There was a large number of enriched Ksuc proteins in the ribosome (1.35e^−14^), cytoplasm (3.52e^−14^), and small ribosomal subunit (2.16e^−05^) in the CC (Fig. 3*B* and supplemental Table S8). This dataset implies that Ksuc proteins were mainly enriched in translation and metabolism processes. To further investigate metabolism-related protein pathways, KEGG metabolic pathway analysis showed that the most enriched Ksuc protein categories were the ribosome (39, 1.50e^−13^), followed by carbon metabolism (37, 3.28e^−08^), glycolysis/gluconeogenesis (23, 4.61e^−08^), pyruvate metabolism (16, 4.59e^−06^), antibiotic biosynthesis (54, 7.88e^−05^), and the citrate cycle (tricarboxylic acid [TCA] cycle; 12, 5.36e^−04^) (Fig. 3*C* and supplemental Table S9). Approximately 36% (n = 131), 25% (n = 90), and 24% (n = 85) of the downregulated succinylated proteins were located in the cytosol, nucleus, and mitochondria, respectively (Fig. 3*D* and supplemental Table S10). Overall, the classification and enrichment data of these annotations indicated that the downregulated succinylated proteins are mainly distributed in energy metabolism and protein-related expression/translation.

### Motif Analysis of Ksuc Peptides

**Supplemental Figure S4:**
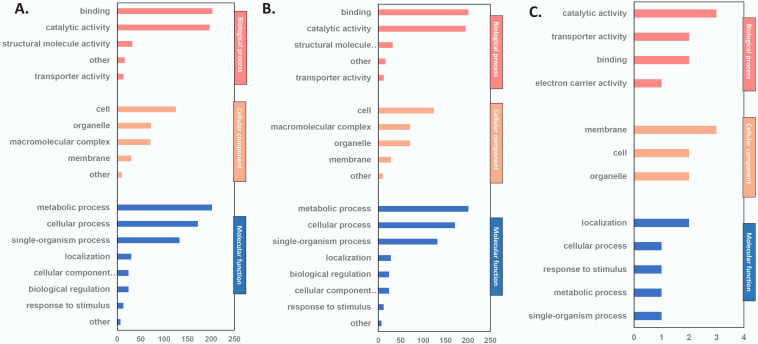
**GO annotation information of identified proteins.***A*, classification of all differentially quantified proteins in GO terms of level 2. *B*, distribution of up-regulated proteins in GO terms of level 2. *C*, distribution of down-regulated proteins in GO terms of level 2.

Motif-X comparison of the six amino acids upstream and downstream of the identified Ksuc sites from the *A. flavus* proteome showed that proline and glutamic acid were present at the +1 and +2 position, respectively (supplemental Fig. S4*A*). The NetSurfP algorithm showed that succinylated lysine was more frequently found in a coil (52.02%), followed by an α-helix (41.3%) and β-sheet (6.85%). Succinylation sites were more enriched on the protein surface (absolute surface accessibility = 90.05) compared with all lysine residues (absolute surface accessibility = 88.34) (supplemental Fig. S4*B*) suggesting that Ksuc may influence the surface properties in a similar manner to other PTMs.

### Protein Interaction Networks of the SKsuc Proteome

**Supplemental Figure S5:**
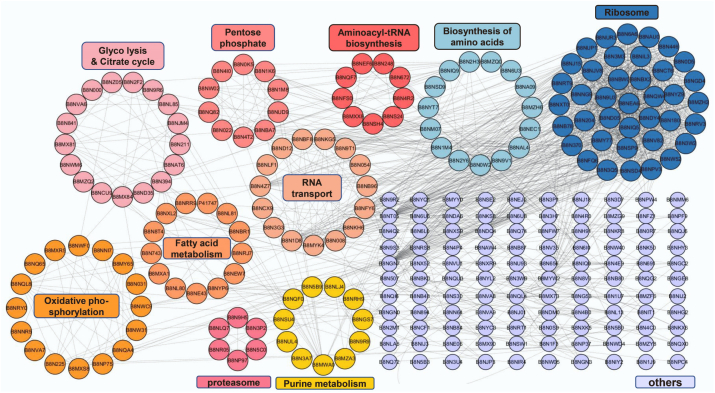
**Analysis of the interaction network of identified succinylated proteins**.

The physical and functional interactions from downregulated proteins were further analyzed using the *A. flavus* PPI database (http://string-db.org/) and Cytoscape to generate a PPI network (supplemental Fig. S5). GO enrichment showed that the functions and activities of the proteins involved in metabolism can be influenced by succinylation, and Ksuc proteins may provide a docking site with adjacent amino acids to influence PPIs or disrupt favorable interactions. Approximately 321 succinylated proteins were constructed and analyzed by KEGG pathway and GO functional annotation with the succinylated proteins forming prominent and highly connected clusters in energy metabolism (supplemental Fig. S5).

### Succinylated Proteins Linked to Metabolic Pathways and Toxin Biosynthesis in *A. flavus*

**Fig. 4:**
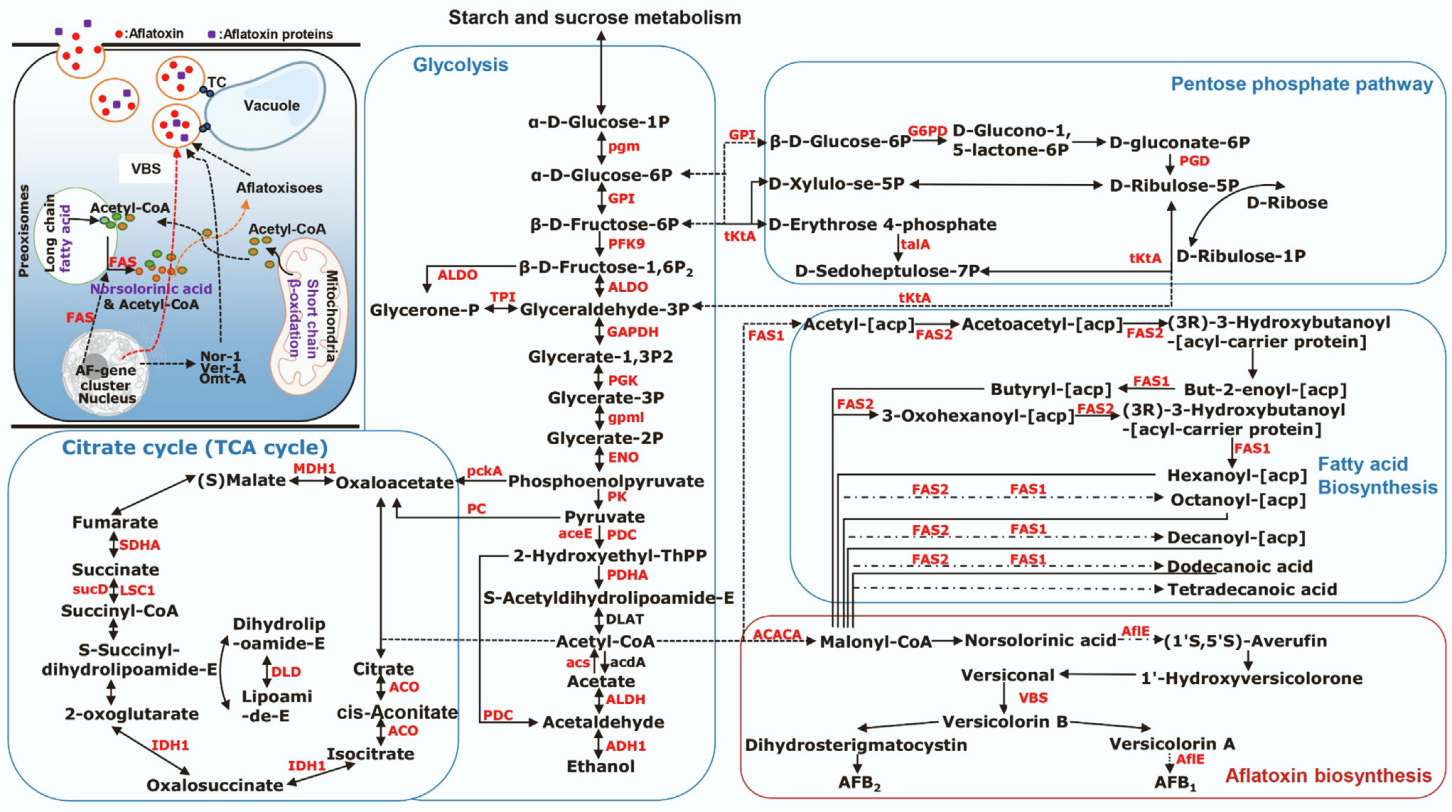
**Overview of the succinylated proteins involved in metabolism and aflatoxin biosynthesis pathways in *Aspergillus flavus*.** The identified succinylated proteins were highlighted in *red*.

Quantified proteins showed significant succinylation differences between high and low toxigenic *A. flavus* strains. The activities of enzymes involved in secondary metabolism and toxin synthesis were localized to vacuoles and vesicles in *Aspergillus* (58). Succinylated metabolic enzymes were mainly enriched in glycolysis, pentose phosphate pathway, fatty acid biosynthesis, citrate cycle (TCA cycle), and AF biosynthesis (Fig. 4). The expression of early AF-related proteins was synthesized on free ribosomes in the cytoplasm. VBS is an oxidoreductase located in the cytoplasm and possibly the Golgi apparatus (59), which can be glycosylated and sense glucose concentration or glucose metabolism to initiate the FadA–cAMP–PKA signaling pathway. Most of the succinylated downregulated proteins involved in energy metabolism were mainly involved in acetyl-CoA metabolism and associated with glycolysis and fatty acid biosynthesis pathways, which was consistent with the metabolic pathway data analysis. At the same time, VBS and AflE were downregulated succinylated proteins directly involved in AF biosynthesis in *A. flavus*. Their enzyme activities were affected by succinylation and are involved in a large number of metabolism-related pathways. Therefore, differences in *A. flavus* toxin production under natural conditions are affected by succinylation.

### Effects of VBS Succinylation on Growth, Conidia, and Sclerotia in *A. flavus*

**Supplemental Figure S6:**
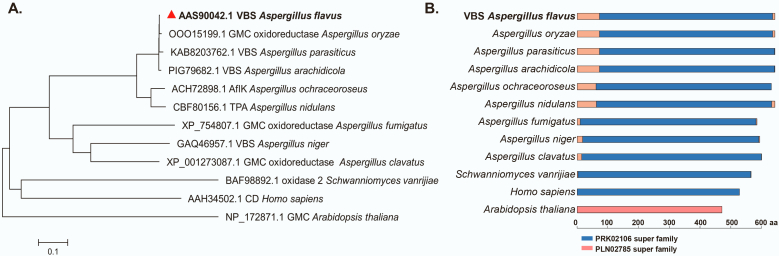
**Phylogenetic and structure analysis of VBS proteins.***A*, phylogenetic analysis of VBS proteins from Aspergillus members and other fungi. Bootstrap values were calculated using the neighbor-joining method with 1000 replicates *A. flavus* was underlined in red. *B*, schematic diagrams of VBS proteins and conserved domain was signed, respectively.

**Supplemental Figure S7:**
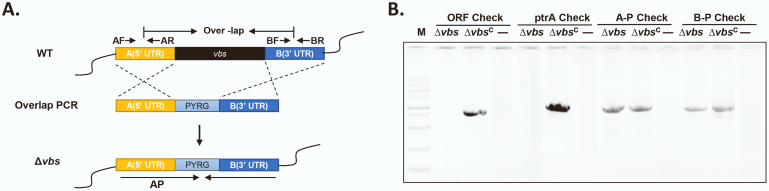
**Schematic for *vbs* deletion and complement.***A*, schematic for replacing the *vbs* gene in *A. flavus* by *pyrg* from Aspergillus fumigates. *B*, PCR diagnosis was carried out to confirm the deletion of genes and primers were listed in supplemental Table S1.

**Supplemental Figure S8:**
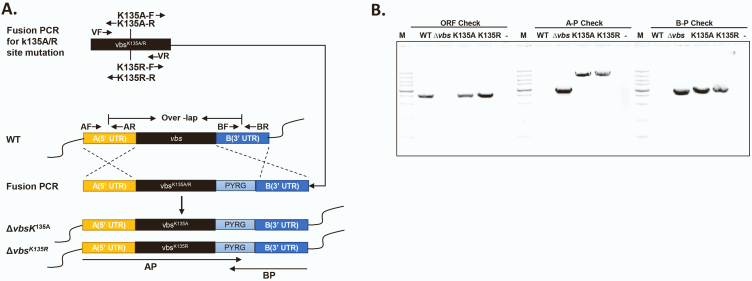
**Schematic for *vbs***^**k135A/R**^**site mutation.***A*, schematic for replacing the *vbs* gene in *A. flavus* by *pyrg* from Aspergillus fumigates and fusion vbsk^135A/R^ fragments. *B*, PCR diagnosis was carried out to confirm mutation of genes. Primers were listed in supplemental Table S1.

**Supplemental Figure S9:**
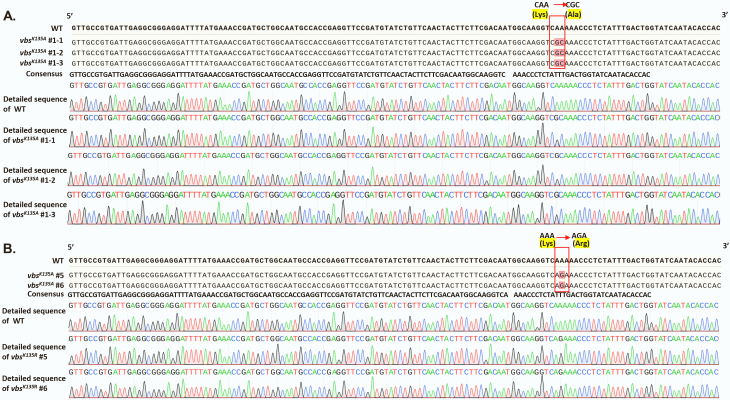
**Identification of site mutation strains.***A*, verification of point mutation *vbs*^k135A^ sequencing from WT and point mutation strains. *B*, verification of point mutation *vbs*^k135R^ sequencing.

Among the succinylated proteins identified, VBS is a downstream protein directly involved in AF biosynthesis. Phylogenetic tree and protein domain analysis indicated that *vbs* is a highly conserved gene in a variety of fungi and suggests that conserved residues may play important roles in VBS function (supplemental Fig. S6). MS analysis observed a reliable succinylation site (K135) on VBS, and this modification may have a key impact on toxin synthesis. The effect of succinylation on *A. flavus* was tested by mutating the *vbs* succinylation modification site and developing knockout *vbs* strains. A *vbs* deletion strain (Δ*vbs*) was constructed using homologous recombination (supplemental Fig. S7*A*) by transforming the plasmid pPTR-*vbs* to prepare the complementation strain (Δ*vbs*^*C*^). The *vbs*^K135A/R^ mutants with *pyrG* fragments were transformed to modify lysine (K) to arginine (R) or alanine (A) to prevent succinylation and maintain the original structure (60). All knockout and complementation strains were confirmed by PCR (supplemental Fig. S7*B*). The Δ*vbs*^K135A^ and Δ*vbs*^K135R^ strains were confirmed by PCR and DNA sequencing (supplemental Figs. S8*B* and S9).

**Fig. 5:**
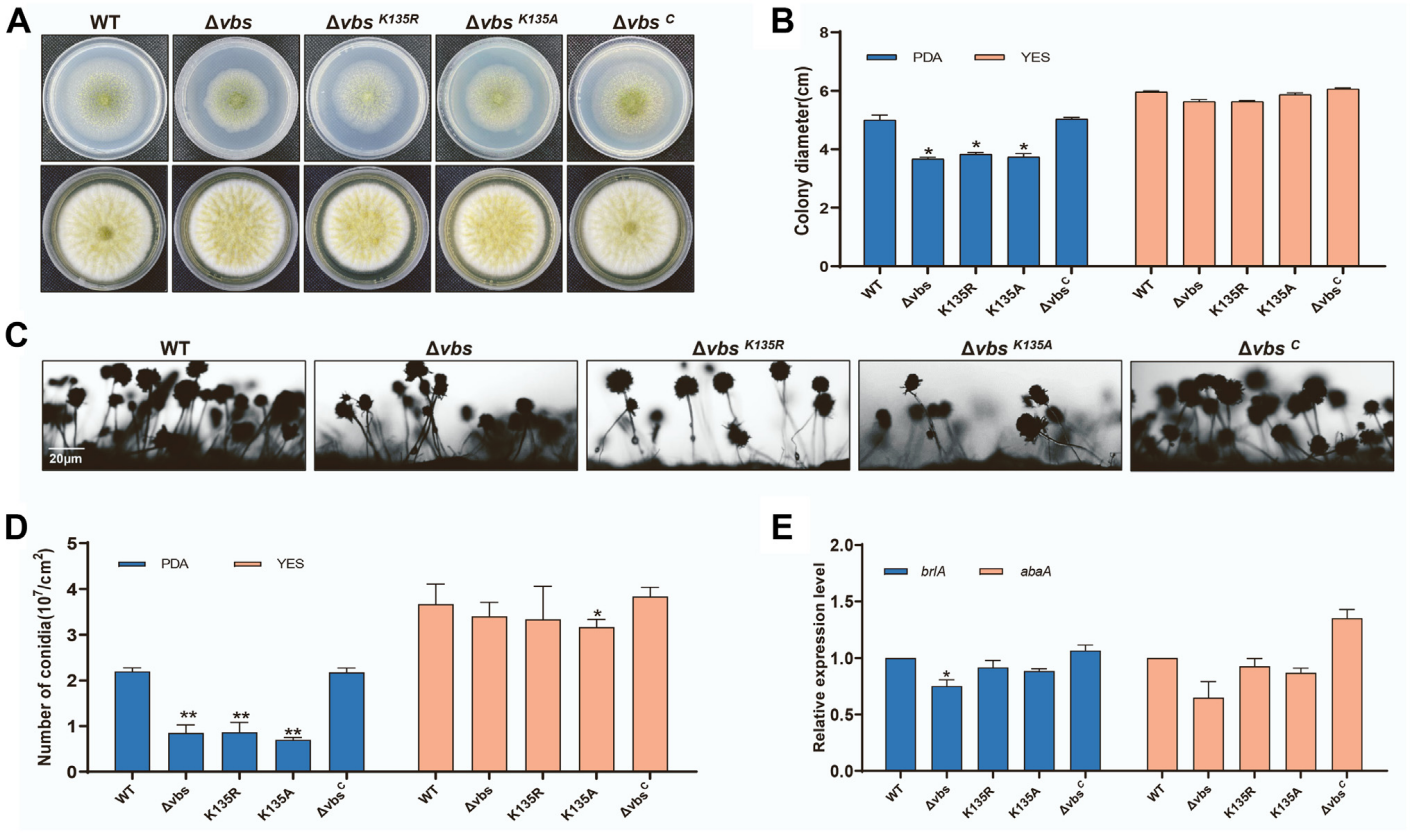
***vbs* gene is involved in fungal vegetative growth and conidiation.***A*, colony morphology of WT, Δ*vbs*, Δ*vbs*^*K135R*^, Δ*vbs*^*K135A*^, and Δ*vbs*^*C*^ strains cultured on potato dextrose agar (PDA) and yeast extract with supplement (YES) plates at 37 °C for 3 days. *B*, growth assays of WT, Δ*vbs*, Δ*vbs*^*K135R*^, Δ*vbs*^*K135A*^, and Δ*vbs*^*C*^ strains. *C*, microscopic observation of asexual development. The conidiophores WT, Δ*vbs*, Δ*vbs*^*K135R*^, Δ*vbs*^*K135A*^, and Δ*vbs*^*C*^ strains were observed after constant light induction for 12 h. Bars represent 20 μm. *D*, conidial amounts of WT, Δ*vbs*, Δ*vbs*^*K135R*^, Δ*vbs*^*K135A*^, and Δ*vbs*^*C*^ strains. Conidia were extracted from each vegetative growth plate and counted by a microscope. *E*, quantitative RT–PCR (qRT–PCR) results showed that two regulatory genes for conidiation were regulated in WT, Δ*vbs*, Δ*vbs*^*K135R*^, Δ*vbs*^*K135A*^, and Δ*vbs*^*C*^. β-actin was used as a reference. The *asterisks* ∗∗ represent a significant difference level of *p* < 0.01.

**Fig. 6:**
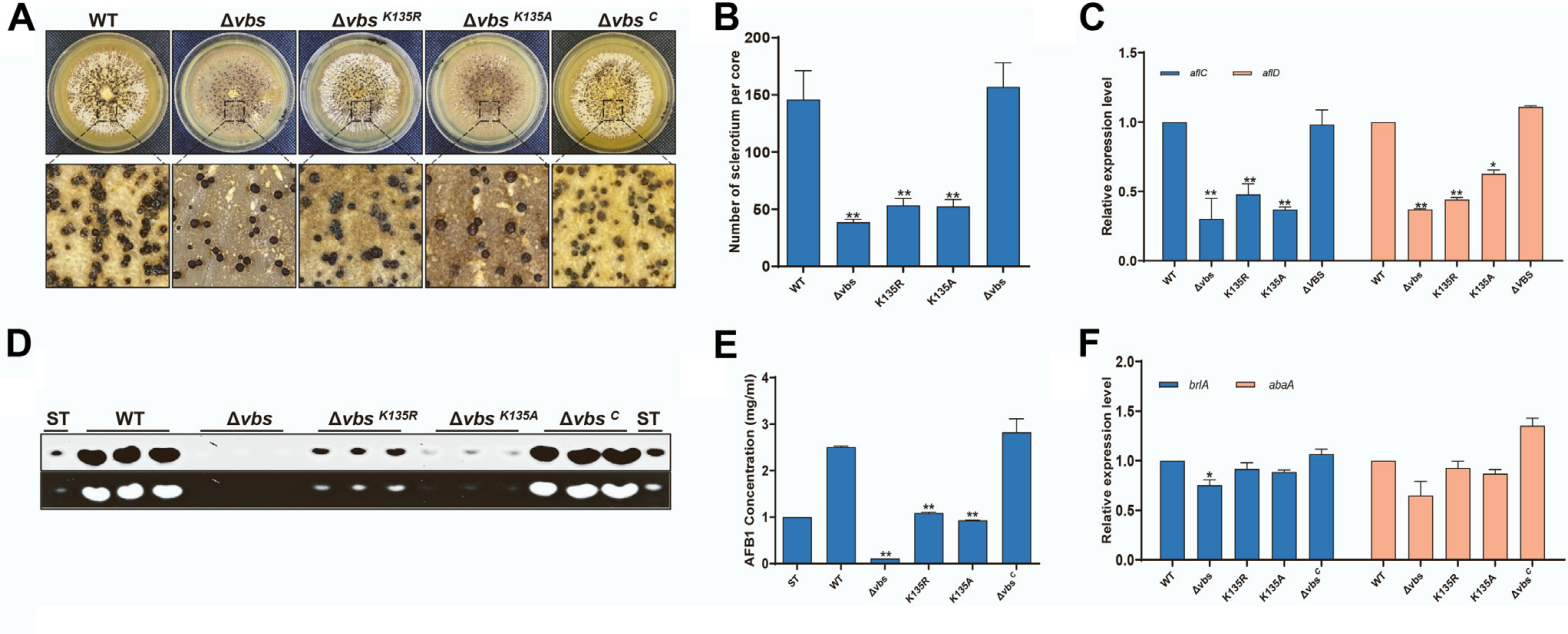
**Effects of *vbs* on sclerotia formation and aflatoxin (AF) biosynthesis in *Aspergillus flavus*.***A*, WT, Δ*vbs*, Δ*vbs*^*K135R*^, Δ*vbs*^*K135A*^, and Δ*vbs*^*C*^ strains were cultured on the yeast peptone dextrose (YPD) plate at 37 °C for 7 days, and the images were taken after the colony was washed with 75% ethanol to expose the sclerotia. *B*, the sclerotia amounts WT, Δ*vbs*, Δ*vbs*^*K135R*^, Δ*vbs*^*K135A*^, and Δ*vbs*^*C*^ strains. *C*, quantitative RT–PCR (qRT–PCR) results showed that two regulatory genes for sclerotia formation were regulated in WT, Δ*vbs*, Δ*vbs*^*K135R*^, Δ*vbs*^*K135A*^, and Δ*vbs*^*C*^. β-actin was used as a reference. *D*, WT, Δ*vbs*, Δ*vbs*^*K135R*^, Δ*vbs*^*K135A*^, and Δ*vbs*^*C*^ strains were cultured on the yeast extract with supplement (YES) plate at 29 °C for 6 days, and TLC plates showed AF production extracted from aforementioned strains. *E*, the AFB_1_ production WT, Δ*vbs*, Δ*vbs*^*K135R*^, Δ*vbs*^*K135A*^, and Δ*vbs*^*C*^. *F*, qRT–PCR results showed that two genes for AF biosynthesis were regulated WT, Δ*vbs*, Δ*vbs*^*K135R*^, Δ*vbs*^*K135A*^, and Δ*vbs*^*C*^. β-actin was used as a reference. The *asterisks* ∗∗ represent a significant difference level of *p* < 0.01 and ∗ represent *p* < 0.05. AFB_1_, aflatoxin B_1_.

The colony phenotype of all strains (WT, Δ*vbs*, Δ*vbs*^*C*^, Δ*vbs*^K135A^, and *vbs*^K135R^) grown on YES and PDA plates was observed to investigate the effects of the nonsuccinylated lysine of VBS on *A. flavus* growth, conidia, and sclerotia (Fig. 5*A*). The colony diameter, number of spores, and microscopic observation of asexual development of the constructed strains were not significantly different from that of WT and Δ*vbs*^C^, and especially similar to WT when cultured in YES media (Fig. 5, *B*–*D*). There was no significant difference in the expression of the two regulatory genes for conidiation in all strains (Fig. 5*E*). Meanwhile, there was significantly less sclerotia in the Δ*vbs* and Δ*vbs*^K135A/R^ strains compared with that of WT and Δ*vbs*^C^ strains (Fig. 6, *A* and *B*). qRT–PCR expression of two genes regulating sclerotia formation (*nsdc* and *nsdd*) was significantly lower in Δ*vbs*^K135A/R^ strains than that of WT and Δ*vbs*^C^ strains (Fig. 6*C*). Therefore, the desuccinylation mimic (Δ*vbs*^K135A/R^) has significant changes compared with the normal strain indicating that succinylation affects the production and development of *A. flavus* sclerotia.

### VBS Succinylation Involved in AF Biosynthesis

The Δ*vbs* strain had almost no detectable AFB_1_ production using TLC assays after culturing in liquid YES medium, whereas the AFB_1_ production of the Δ*vbs*^K135A^ and *vbs*^K135R^ strains was significantly lower than that of the WT and Δ*vbs*^C^ strains (Fig. 6, *D* and *E*). This suggests that K135 succinylation of VBS is an important regulator of AF synthesis. qRT–PCR results showed that the expression level of two genes involved in AF biosynthesis (*aflC* and *aflD*) significantly decreased in Δ*vbs*, Δ*vbs*^K135A^, and *vbs*^K135R^ compared with WT and Δ*vbs*^C^ strains (Fig. 6*F*). Therefore, the differences in AF production among WT, Δ*vbs*, Δ*vbs*^C^, Δ*vbs*^K135A^, and *vbs*^K135R^ were caused by abnormal expression of AF-related genes. Taken together, these results suggest that VBS is an essential protein for *A. flavus* toxin production, and its succinylation is a key factor in regulating toxin synthesis.

### Effects of VBS Succinylation in Seed Pathogenicity

**Fig. 7:**
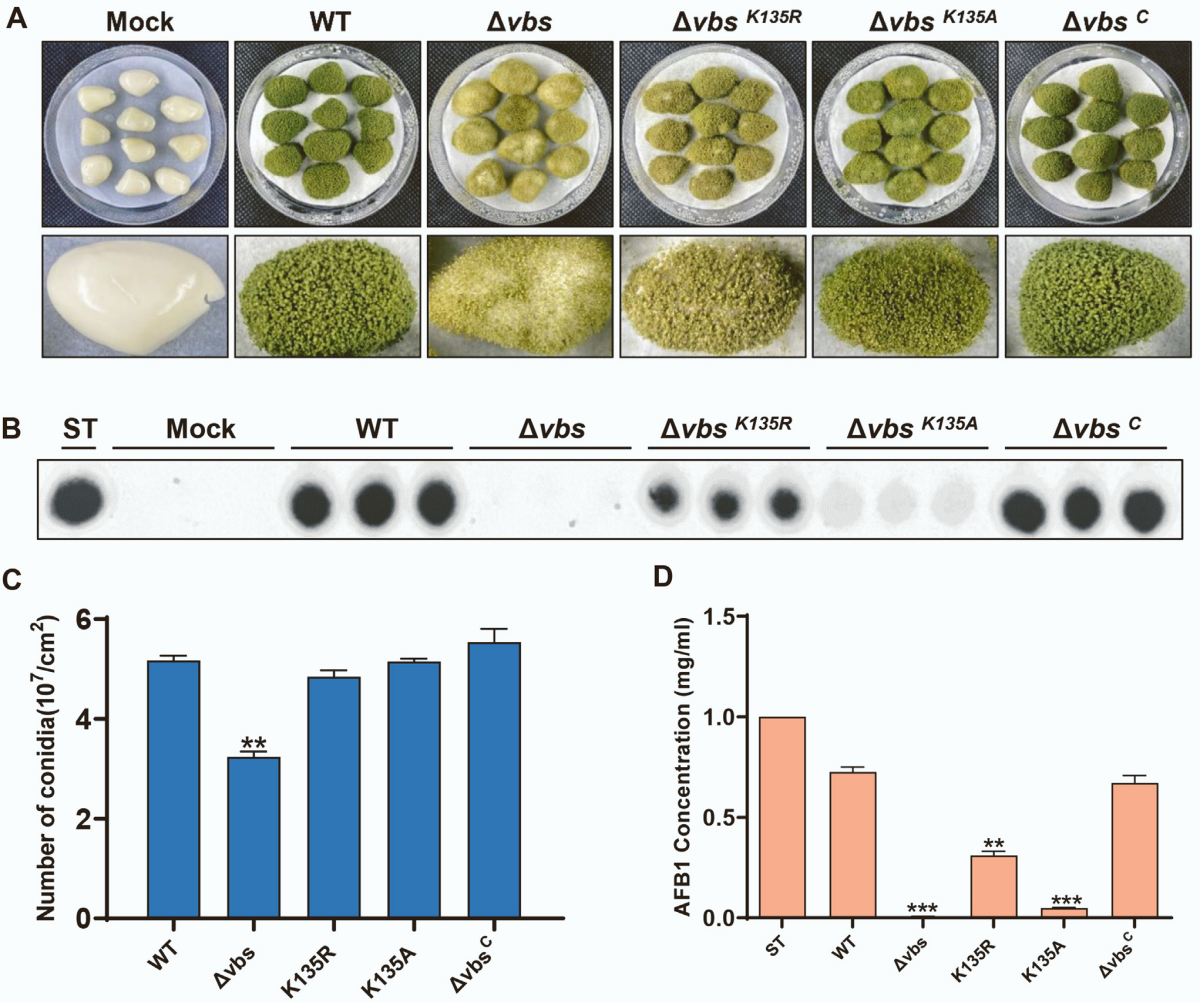
**Pathogenicity analysis of WT, Δ*vbs*, Δ*vbs***^***K135R***^**, Δ*vbs***^***K135A***^**, and Δ*vbs***^***C***^**strains.***A*, morphology of *Aspergillus flavus* on peanut after 5 days of inoculation. Mock means seed inoculated with sterile water as control. *B*, conidia production from infected seeds. *C*, TLC measurement of AFB_1_ extracted from the infected seeds. *D*, quantification analysis of AFB_1_ from the infected seeds. The *asterisks* ∗∗∗ represent a significant difference level of *p* < 0.001 and ∗∗ represent  *p* < 0.01. AFB_1_, aflatoxin B_1_.

*A. flavus* is one of the main invasive fungi, and its infectivity is an important part of its functional research. We inoculated *A. flavus* spores onto sterilized peanut cotyledons to determine plant infectivity. The Δ*vbs* strain had the least number of conidia relative to other strains, whereas Δ*vbs*^K135A^ and *vbs*^K135R^ had no significant differences in the number of conidia compared with WT and Δ*vbs*^C^ (Fig. 7, *A* and *C*). At the same time, Δ*vbs*-infected peanut cotyledons contained almost undetectable AF production, and Δ*vbs*^K135A^ and *vbs*^K135R^ produced significantly reduced AF production in infected peanut cotyledons compared with WT and Δ*vbs*^C^ (Fig. 7, *B* and *D*). Our study showed that VBS succinylation plays an important role in AF production of toxins in plants infected with *A. flavus*.

## Discussion

Ksuc is the transfer of a succinyl group to a lysine residue of a substrate protein (61). The involvement of sirtuin 5 desuccinylase in various key metabolic cellular processes is now widely recognized (62). In fungi, biosynthetic production of SMs is an important part of fungal functional studies, and AF has been one of the most studied SMs (63). In our previous study, *A. flavus* standard strain 3357 was used to explore the AF biosynthesis under the treatment of sodium succinate; we first discover that the Ksuc is involved in the secondary metabolic pathway and AF biosynthesis of *A. flavus* (64). In order to further explore the regulatory function and mechanism of succinylation in AF biosynthesis, the study of measuring Ksuc levels under different AF yields in natural conditions is relatively blank. Thus, we perform a TMT-labeled quantitative lysine succinylome to comprehensively report the changes in Ksuc levels between high and low AF production strains of *A. flavus* isolated under natural conditions. From our quantitative global proteomics data, we noticed that the upregulated proteins in the high AF yield group were mostly enriched in carbon-related metabolism, whereas the downregulated proteins were enriched in oxidative phosphorylation; through subsequent functional analysis, we found that the upregulated proteins involved in carbon-related metabolism had a direct relationship with AF production (48).

In this study, by comparing the differences in succinylation levels of the two strains and exploring the relationship between succinyl modification and AF synthesis, our succinylome data identified 1240 Ksuc sites in 768 proteins, among which 1103 Ksuc sites in 685 proteins were quantified. In particular, we were surprised to find that Ksuc were significantly downregulated in a large number of pathways (ribosome, mitochondrion, glycolysis, pentose phosphate pathway, fatty acid biosynthesis, peroxisome, citrate cycle [TCA cycle], and AF biosynthesis) associated with *A. flavus* SMs, and some are directly involved in AF biosynthesis. In detail, among the quantified proteins, eight Ksuc sites in eight proteins were upregulated and 502 Ksuc sites in 360 proteins were downregulated in H-AFs/L-AFs (Fig. 2*D*). Among these metabolic pathways, the glycolysis, pentose phosphate, fatty acid biosynthesis, citrate cycle (TCA cycle), and AF biosynthesis are related to acyl-CoA, the important precursor for AF synthesis (22), indicating that succinylation can severely regulate the formation of toxin-related SMs. In addition, succinylation in yeast or *Escherichia coli* cells is caused by succinyl-CoA through the TCA cycle since it is a succinyl donor for lysine, and succinyl-CoA concentration affects global succinylation levels (41). We found that most of the enzymes involved in the TCA cycle undergo Ksuc (supplemental Fig. S5), which correlates with previous research (62, 65), suggesting that Ksuc may have an important regulatory role in TCA cycle–related protease activity. Moreover, AF synthesis is negatively correlated with the level of succinylation of metabolism-related proteins in H-AFs/L-AFs. Interestingly, Yang *et al.* (66) found that *A. flavus* treated with sodium succinate exhibited greater total protein succinylation but showed significantly decreased AF production, which indirectly suggests that AF synthesis is affected by the overall negative regulation of succinylation. AF biosynthesis involves the activity of at least 17 enzymes encoded by approximately 25 or more genes clustered in a 75 kb region on one chromosome (58). AFs are polyketide-derived furanocoumarins, which are initially formed by conversion of acetyl-CoA to malonyl-CoA catalyzed by acetyl-CoA carboxylase, followed by Fas-1- and Fas-2-driven catalysis to form the starting unit hexanoate (67). Succinylation of acetyl-CoA carboxylase, Fas-1, and Fas-2 was all significantly downregulated in H-AFs. These results suggest that Ksuc is an important PTM involved in the secondary metabolic process of AF synthesis, which may directly regulate the structure or activity of enzymes involved in the initial stage of AF synthesis.

VBS and AflE have key catalytic roles in versicolorin B and norsolorinic acid synthesis, respectively (68). Our quantitative data demonstrated that the VBS and AflE succinylation levels in H-AFs are lower than that of L-AFs. In addition, the levels of succinylation of proteins associated with AF production were significantly downregulated. Therefore, we hypothesized that the enzyme activity in the AF biosynthetic pathway is negatively regulated by succinylation. Knockout and site mutation of strains of VBS protein was performed to explore the function of Ksuc in *A. flavus* development and AF biosynthesis. Sclerotia development and AF production of Δ*vbs* and Δ*vbs*^K135A/R^ were significantly reduced compared with the WT (Fig. 6). The infectivity of the mutant *A. flavus* strains did not change since there was no difference in the number of conidia; however, AF production was significantly reduced after infection in all mutant strains. The results showed that lysine residues mutated to either A or R affected the phenotype, and the mutation to R was only partially affected, whereas the mutation to A completely lost the relevant phenotype. Since the K to R mutation was mimicked by similar structures containing positive charges and the A mutation was mimicked by the site-removed state, this result also indirectly indicated that succinylation does affect the function of VBS.

In summary, 1240 Ksuc sites in 768 proteins, among which 1103 Ksuc sites in 685 proteins were quantified, and the majority of proteins involved in AF biosynthetic pathway were downregulated in high AF-producing strains suggesting that Ksuc may be an important regulatory mechanism in affecting AF production in the naturally isolated *A. flavus* strains. In function analysis, comparison of the Ksuc levels of the immunoprecipitated VBS from WT VBS and its mutants (K135A and K135R) was done. We further confirmed that the K135 is critical for VBS activity, whereas succinylation of K135 is a key factor in maintaining sclerotia and AF production. Overall, our quantitative lysine succinylome has comprehensively and deeply reported the regulation mechanism between Ksuc and AF synthesis in *A. flavus*. In addition, the differences in Ksuc of naturally isolated *A. flavus* strains also indicated that the natural environment can affect the level of succinylation, thereby regulating AF synthesis, providing clues for the impact of environmental stress on Ksuc.

## Data Availability

All resulting raw data were uploaded to the ProteomeXchange repository with the dataset identifier PXD038357.

## Supplemental Data

This article contains supplemental data.

## Conflict of interest

The authors declare no competing interests.
