# Supplementary material for: Lysine Succinylation of VBS Contributes to Sclerotia Development and Aflatoxin Biosynthesis in Aspergillus flavus
Source: Mol Cell Proteomics. 2022 Dec 22;22(2):100490. doi: 10.1016/j.mcpro.2022.100490 (PMC9879794; doi:10.1016/j.mcpro.2022.100490)
Supplement: Supplemental Table S1 [file mmc1.docx]

**Table. S1 Primers used for VBS deletion and site mutation.**

| **Primer Name** | **Sequence (5‘-3’)** | **application** |
| --- | --- | --- |
| D-AF | CGATTCCTCGAGGGAAAAGACT | 5' UTR of *vbs* |
| D-AR | TGAAGAGCATTGTTTGAGGCACGGTAGCCGCTGATAATACATTG |  |
| D-BF | GCATCAGTGCCTCCTCTCAGAAAGTTATATGTGTTACATACCGTTCTCG | 3' UTR of *vbs* |
| D-BR | CGAAGGTGTGGAAAGACCCTCA |  |
| *pyrG* F | GCCTCAAACAATGCTCTTCACCC | Fumigatus *pyrG* |
| *pyrG* R | GTCTGAGAGGAGGCACTGATGC |  |
| D-overlap-F | GCGACTGGTGCTTCGCAG | Fusion deletion fragment |
| D-overlap-R | GTTCTGCGCCTGTGAATG |  |
| *vbs*O-F | ATGGGACGAAACTGGTTCC | *vbs* ORF check |
| *vbs*O-R | CTACTGCTCAGCCATCATTTC |  |
| *vbs* 70-F | AATCCGACCATCTTGTCC | *vbs* vertification |
| *vbs* 873-R | AGGTTGCCATTGGAGAAG |  |
| *pyrG*801-R | CAGGAGTTCTCGGGTTGTCG | *pyrg* vertification |
| *pyrG*1020 F | ATCGGCAATACCGTCCAGAAGC |  |
| *vbs*com-F | CTATGACCATGATTACGCCAAGCTTAGGATCAGATCTGGG | completement fused to vector |
| *vbs*com-R | CCAGTGAATTCGAGCTCGGTACCTGATAGTTTCATCGCC |  |
| M-AF | ATCACTTTAGGATAGGAAGCGTG | 5' UTR of *vbs* to *vbs*^K135R^ |
| M-K135R-R | GTCAAATAGAGGGTTT CTGACCTTGCCATTGTCGAAG |  |
| M-K135R-F | CTTCGACAATGGCAAGGTCAGA AACCCTCTATTTGAC | *vbs*^K135R^ to *vbs* terminator |
| *vbs* -R | GGGTGAAGAGCATTGTTTGAGGC CTACTGCTCAGCCATC |  |
| M-BF | GCATCAGTGCCTCCTCTCAGACATGAGGGTGGATC | 3' UTR of *vbs* |
| M-BR | GTTCTGCGCCTGTGAATG |  |
| M-K135A-R | GTCAAATAGAGGGTTTGC GACCTTGCCATTGTCGAAG | 5' UTR of *vbs* to *vbs*^K135A^ |
| M-K135A-F | CTTCGACAATGGCAAGGTCGCAAACCCTCTATTTGAC |  |
| M-overlap-F | CCGTTCGCAGTTACCCTTTATT | site-mutation fusion PCR |
| M-overlap-R | CACTGGGATAAAACCTTCAC |  |
