# Supplementary material for: Lysine Succinylation of VBS Contributes to Sclerotia Development and Aflatoxin Biosynthesis in Aspergillus flavus
Source: Mol Cell Proteomics. 2022 Dec 22;22(2):100490. doi: 10.1016/j.mcpro.2022.100490 (PMC9879794; doi:10.1016/j.mcpro.2022.100490)
Supplement: Supplemental Table S2 [file mmc2.docx]

**Table S2. Primers used for qRT-PCR**

| **Primer Name** | **Sequence (5‘-3’)** |
| --- | --- |
| β-actin-Q | ACGGTGTCGTCACAAACTGG |
|  | CGGTTGGACTTAGGGTTGATAG |
| aflD | GTGGTGGTTGCCAATGCG |
|  | CTGAAACAGTAGGACGGGAGC |
| aflC | GGTGGTGGACAAAGCGTTAGT |
|  | CGTAGTAGCAGCAGCCTTGG' |
| brlA | GCCTCCAGCGTCAACCTTC |
|  | TCTCTTCAAATGCTCTTGCCTC |
| abaA | CACGGAAATCGCCAAAGAC |
|  | TGCCGGAATTGCCAAAG |
| nsdC | GCCAGACTTGCCAATCAC |
|  | CATCCACCTTGCCCTTTA |
| nsdD | GGACTTGCGGGTCGTGCTA |
|  | AGAACGCTGGGTCTGGTGC- |
